# Supplementary material for: Insight into the Structural Dynamics of the Lysenin During Prepore-to-Pore Transition Using Hydrogen–Deuterium Exchange Mass Spectrometry
Source: Toxins (Basel). 2019 Aug 7;11(8):462. doi: 10.3390/toxins11080462 (PMC6722932; doi:10.3390/toxins11080462)
Supplement: Supplementary file 1 [file toxins-11-00462-s001.pdf]

Supplementary Materials: Insight into the Structural Dynamics of the Lysenin During Prepore-to-Pore Transition Using Hydrogen-Deuterium Exchange Mass Spectrometry

Magdalena Kulma, Michał Dadlez and Katarzyna Kwiatkowska

Supplementary Material and Methods

Measurement of Lysenin Adsorption at the Water–Argon Interface

Surface pressure was measured at 21 ± 1°C for 2000 sec, and monitored using a NIMA Technology tensiometer, model PS3 (Coventry, UK). The water subphase was buffered with 30 mM Tris–HCl (pH 7.5). Recombinant lysenin proteins were injected into the aqueous sub-phase at a concentration range of 0.5–40 μM.

Lytic Activity of Lysenin Against HEK293 Cells

To analyze the pore-forming activities of lysenin^{WT} and lysenin$^{V88C/Y131C}$ in mammalian cells, we measured the plasma membrane's permeability to calcein as previously described [1], with some modification. HEK293 cells in a 12-well plate (0.5 × 10^6 cells/500 μL of TBS) were incubated for 1 h at 37 °C in darkness with 1 μM calcein-AM (Sigma). After washing with TBS, cells were incubated with various concentrations of lysenin^{WT} and lysenin$^{V88C/Y131C}$ with or without 10 mM DTT. After centrifugation (500 × *g* for 5 min), we measured the amount of calcein released using a JASCO FP 6500 spectrofluorimeter at an Ex/Em of 460/517 nm. The calcein efflux induced by recombinant proteins was expressed as the percentage of the maximal efflux determined in the presence of Triton X-100.

Blue Native-Polyacrylamide Gel Electrophoresis (BN-PAGE)

Lipid-bound samples were prepared according to procedure described for HDX measurements. Before application onto polyacrylamide gradient 4–20% PROTEAN TGX Precast Protein Gels (Bio-Rad) protein samples were supplemented with blue native sample buffer to final concentrations of 10% glycerol, 0.5 % bromophenol blue, and 31 mM Tris-HCl, pH 6.8. Protein were visualized by colloidal Coomassie G-250 staining.

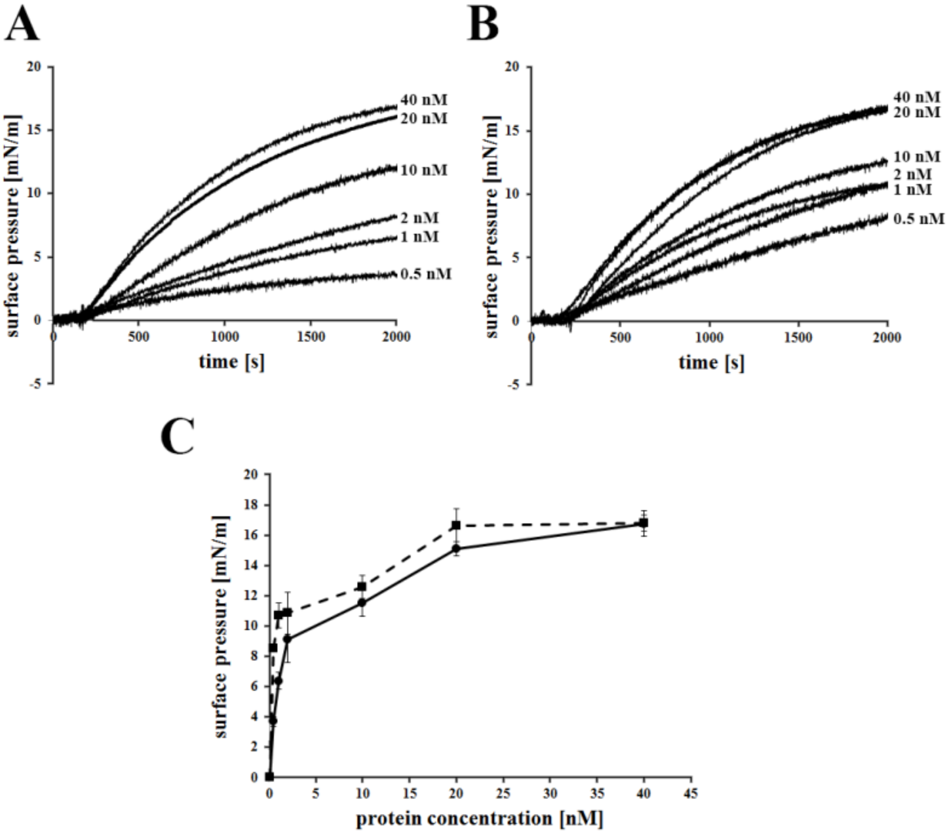

Figure S1. Adsorption of lysenin at the argon–water interface. (**A**,**B**) Surface pressure changes caused by various concentrations of lysenin^{WT} (**A**) and lysenin$^{V88C/Y131C}$ (**B**) in a time-dependent manner. Plots are representative of three independent measurements. (**C**) Maximum pressure value caused by lysenin adsorption at the argon–water interface for 2000 sec as a function of the concentrations of lysenin^{WT} (*solid line*) and lysenin$^{V88C/Y131C}$ (*dashed line*) in the water sub-phase. Data are presented as the mean ± S.E.M. from three experiments.

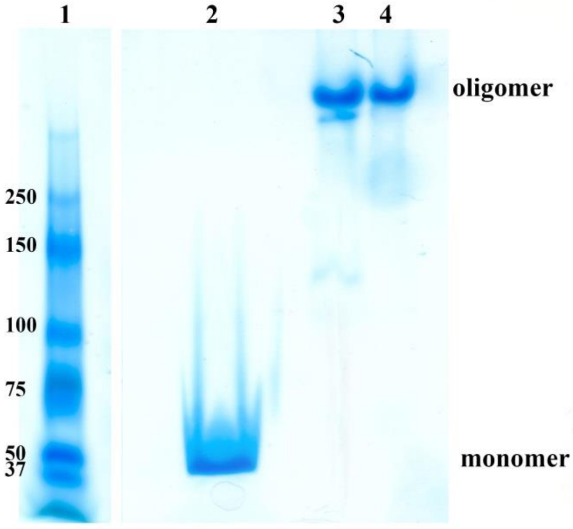

Figure S2. Analysis of oligomer formation by lysenin^{WT} and lysenin$^{V88C/Y131C}$ under native conditions using blue native PAGE. Blue native gel electrophoresis reveals that membrane-bound lysenin^{WT} and lysenin$^{V88C/Y131C}$ mainly exist in one oligomeric state. Lane 1-marker; Lane 2- soluble lysenin^{WT};

Lane 3- lysenin$^{V88C/Y131C}$ bound to SM-containing liposomes; Lane 4- lysenin^{WT} bound to SM-containing liposomes.

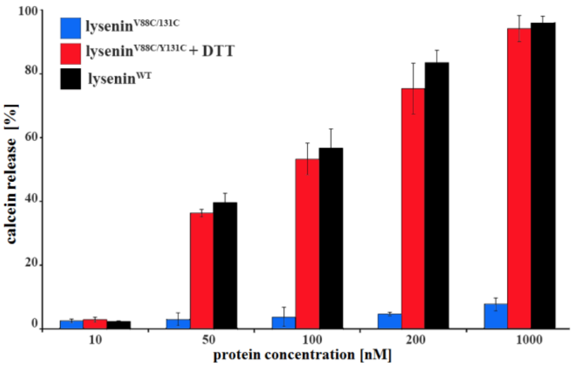

Figure S3. Lytic activity of lysenin. Calcein released from HEK293 cells induced by lysenin^{WT} (black bars), lysenin$^{V88C/Y131C}$ with 10 mM DTT (red bars), or lysenin$^{V88C/Y131C}$ without 10 mM DTT (blue bars), after a 1-hour incubation at 37 °C. Data are presented as the mean ± S.E.M. from three experiments.

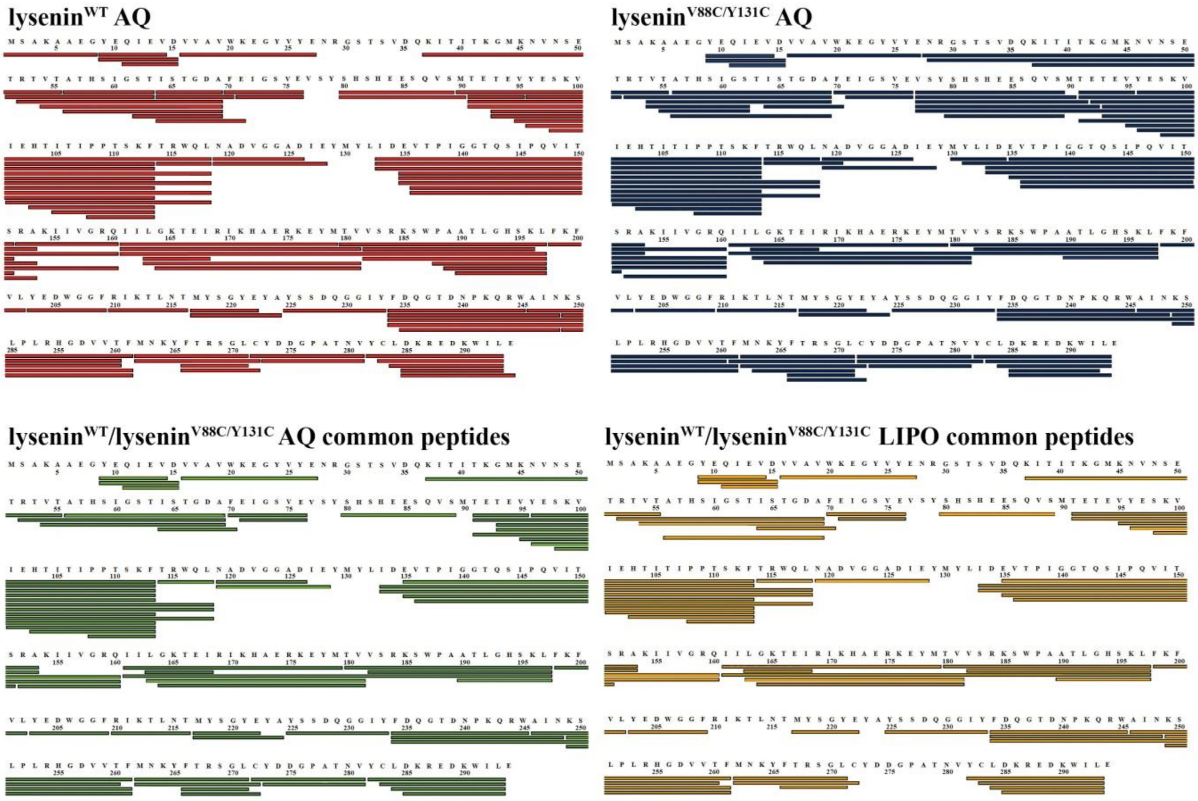

Figure S4. Map of the peptides used to monitor the HDX of lysenin^{WT} and lysenin$^{V88C/Y131C}$. Red and blue, respectively, indicate the sequence coverage of the identified peptic peptides of lysenin^{WT} and lysenin$^{V88C/Y131C}$ in aqueous solution. Green and yellow, respectively, indicate the sequence coverage of the identified peptic peptides common to lysenin^{WT} and lysenin$^{V88C/Y131C}$in aqueous solution and bound to liposomes.

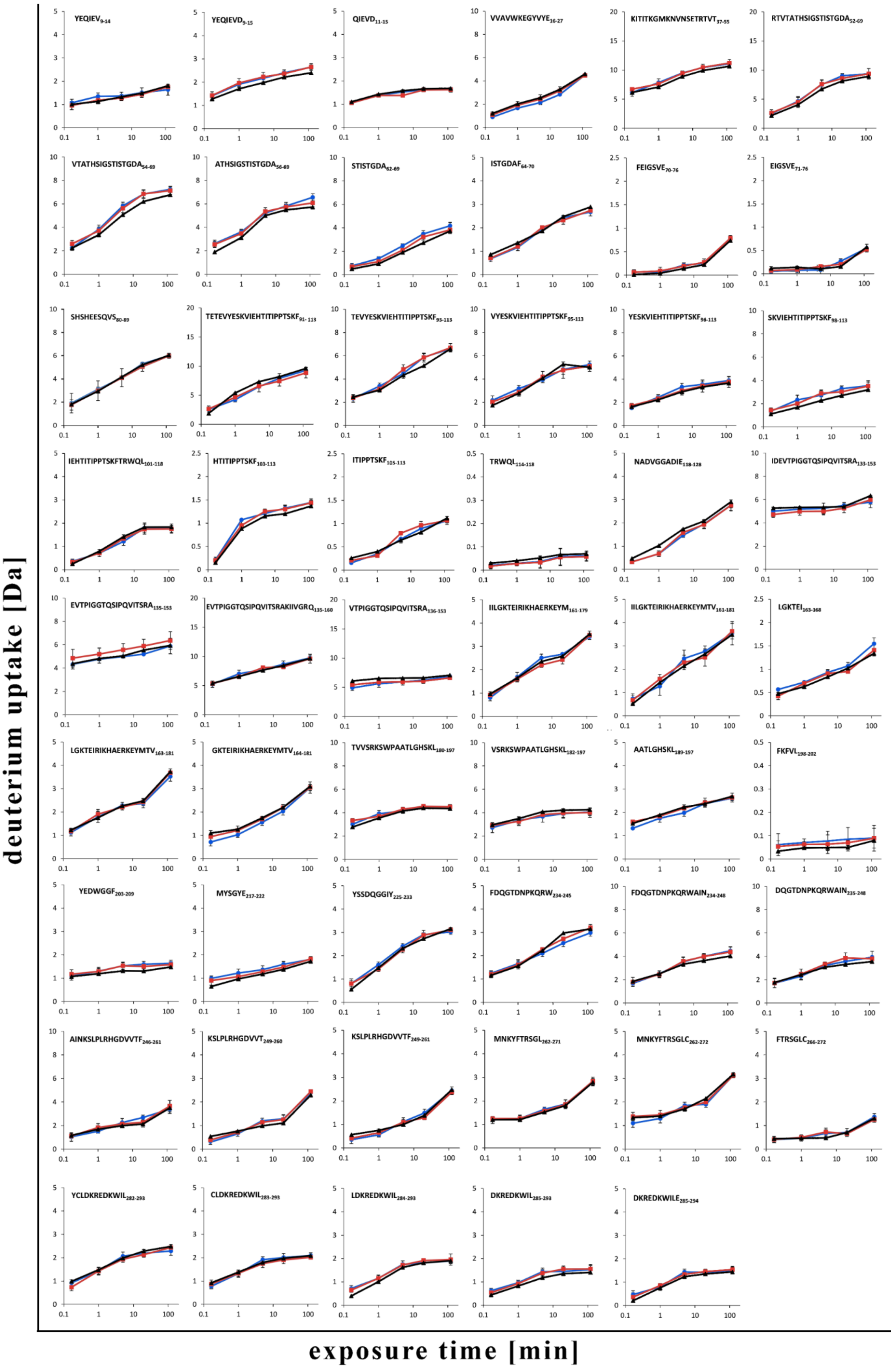

Figure S5. Effects of the V88C/Y131C mutation on the deuterium uptake of lysenin in aqueous solution. Plots show the relative deuterium uptake for peptides in lysenin^{WT} (black) versus lysenin$^{V88C/Y131C}$ without DTT (blue) and lysenin$^{V88C/Y131C}$ supplemented with 10 mM DTT (red) on a linear time scale. The y-axis error bars show standard deviations calculated from at least three independent experiments.

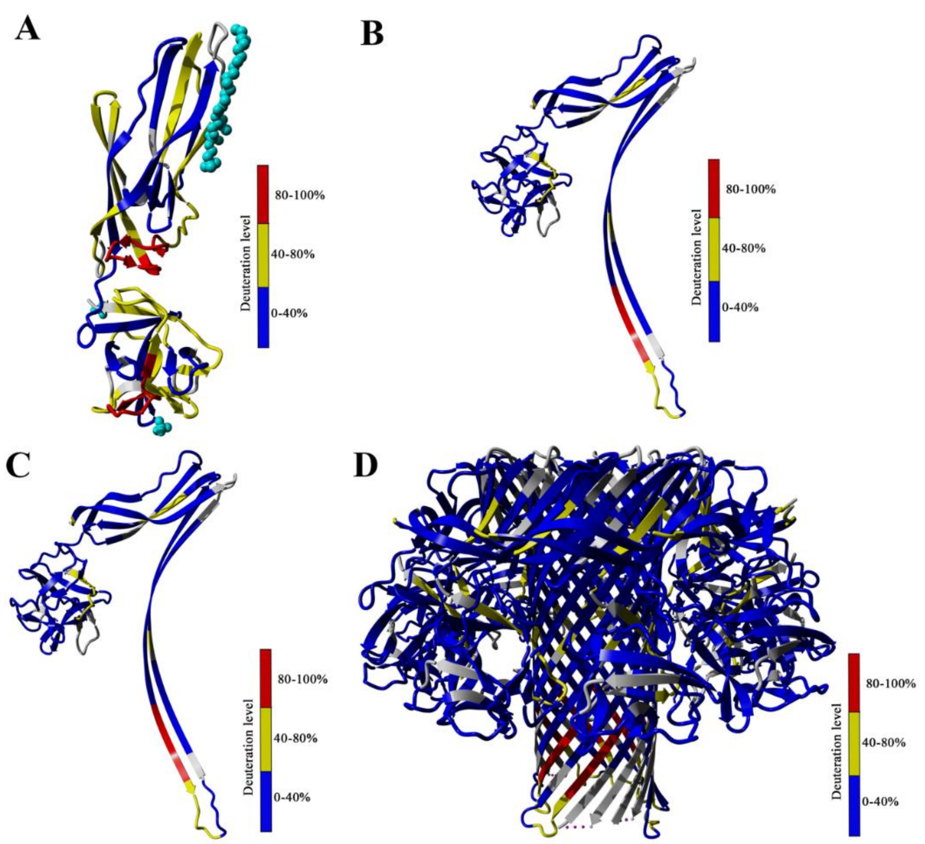

Figure S6. The overlay of HDX results of peptides on the crystal structures of lysenin. The color legend indicates the level of deuterium exchange after 20 min: red, deuterium exchange in the range of 80–100%; yellow, 40–60%; blue, 0–40%; gray, regions not covered by the data. (**A**) Results for lysenin$^{V88C/Y131C}$ bound to SM-containing liposomes without DTT overlaid on the structure of monomeric lysenin bound to SM (PDB ID: 3ZXG); results for lysenin$^{V88C/Y131C}$ bound to SM-containing liposomes in the presence of DTT (**B**) and lysenin^{WT} bound to SM-containing liposomes (**C**) overlaid on the protomer from the lysenin pore (PDB ID: 5EC5); (**D**) results obtained for lysenin^{WT} bound to SM-containing liposomes overlaid on the lysenin pore structure (PDB ID: 5EC5).

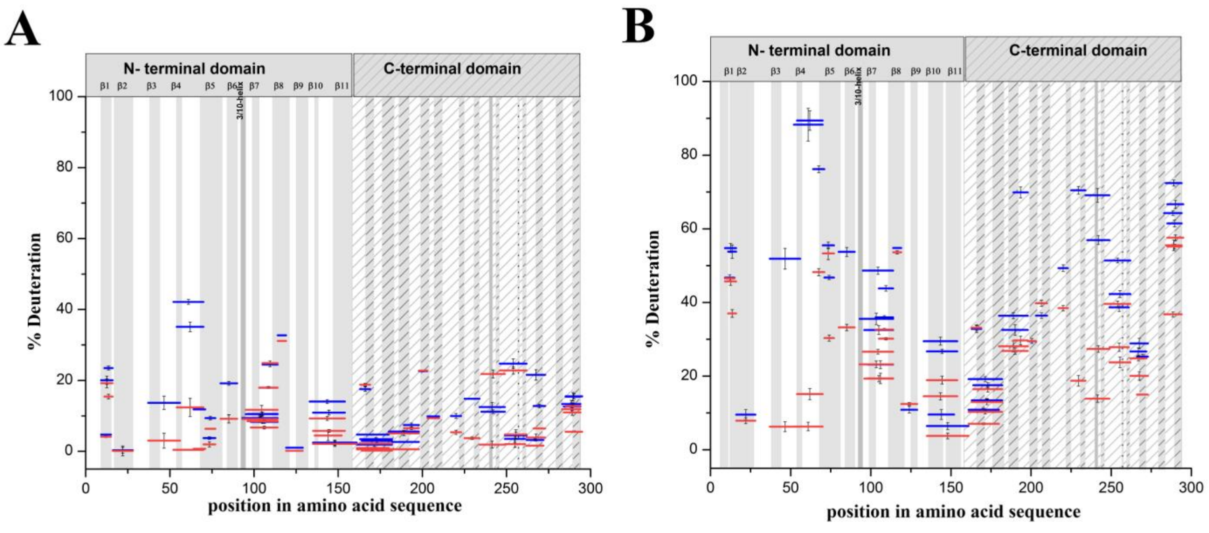

Figure S7. Time-dependent hydrogen-deuterium exchange patterns of lysenin$^{V88C/Y131C}$ upon binding to liposomes. Samples of lysenin$^{V88C/Y131C}$ without DTT (blue), and lysenin$^{V88C/Y131C}$ supplemented with 10 mM DTT (red) bound to SM-containing liposomes were incubated with deuterium buffer

for 10 s (**A**) or 20 min (**B**). Peptides are represented with horizontal bars indicating their lengths and positions in the lysenin amino acid sequence (horizontal axis).

HHHHHHSSGLVPRGSHMASMTGGQQMGRGSMSAKAAEGYEQIEVDV
VAVWKEGYVYENRGSTSVDQKITITKGMKNVNSETRTVTATHSIGSTIS
TGDAFEIGSVEVSYSHSHEESQVSMTETEVYESKVIEHTITIPPTSKFTR
WQLNADVGGADIEYMYLIDEVTPIGGTQSIPQVITSRAKIIVGRQIILGK
TEIRIKHAERKEYMTVVSRKSWPAATLGHSKLFKFVLYEDWGGFRIKT
LNTMYSGYEYAYSSDQGGIYFDQGTDNPKQRWAINKSLPLRHGDVVTF
MNKYFTRSGLCYDDGPATNVYCLDKREDKWILEVVG

Figure S8. Amino acid sequence of recombinant lysenin. Lysenin sequence is marked in black; mutation sites (V88 and Y131) are underlined and marked in red; N-terminal His tag, thrombin cleavage site, internal T7 epitope tag derived from pET28 vector are marked in blue.

Supplemetary References

1. Kulma, M.; Kwiatkowska, K.; Sobota, A. Raft coalescence and FcγRIIA activation upon sphingomyelin clustering induced by lysenin. *Cell. Signal.* **2012**, *24*, 1641–1647, doi:10.1016/j.cellsig.2012.04.007.
